# A retrospective analysis of caries treatment and development in relation to assessed caries risk in an adult population in Sweden

**DOI:** 10.1186/1472-6831-14-126

**Published:** 2014-10-17

**Authors:** Ulf Söderström, Ingegerd Johansson, Karin Sunnegårdh-Grönberg

**Affiliations:** Department of Public Dental Service, County Council of Västerbotten, Umeå, Sweden; Department of Odontology/Cariology, Umeå University, Umeå, Sweden

**Keywords:** Caries, Caries risk, Caries prevention, Public dental care

## Abstract

**Background:**

The Public Dental Service of Västerbotten County (Sweden) recommends using population-based prevention strategies combined with an individual strategy for high-risk patients to manage caries. To facilitate this management strategy, all patients are evaluated for their risk of developing caries in the coming year using defined criteria. Using caries risk scoring over a seven-year period, the present study evaluates prophylactic measures, caries development, and non-operative treatments in adult patients.

**Methods:**

From all adult patients (25–65 years; n = 76 320) scored with a high caries risk in 2005 (baseline) and with a dental visit in 2011, 200 subjects were randomly selected. In addition, an equally sized control group with a no/low caries risk was selected. Information concerning dental status, counselling, treatments, visits, and costs were retrieved from dental records.

**Results:**

Over the seven-year study period, subjects with high caries risk had significantly higher caries incidence in spite of shorter recall intervals, more dental appointments, and higher costs for dental care than subjects with no/low caries risk. Non-operative measures, such as additional fluoride and individual counselling on diet at baseline (2005), was higher in the high caries risk group, whereas information about basic prophylaxis and counselling on oral hygiene showed only small differences. The frequency of non-operative measures given during the seven-year study period to patients in the high caries risk group is considered to be remarkably low and improvement, determined as reclassification from high to no/low caries risk from 2005 to 2011, was seen in only 13% of the participants.

**Conclusions:**

This study formulated two major conclusions. First, adult patients with high or no/low caries represent different populations, that each contain distinct subpopulations, those who improve/impair or maintained their caries risk and disease progression. These groups need different strategies in disease treatment. Second, preventive measures and non-operative treatments were associated with improvements in caries risk and maintenance, but the extent to which such treatments were given to high caries risk subjects was unacceptably low. Improved adherence to the guidelines for caries treatment may reduce caries risk, visits to dental clinics, and costs for the patients.

## Background

Caries is the most common lifestyle-influenced disease in children and adults worldwide [1, 2]. Although preventable, the treatment of primary and secondary caries, and replacement of restorations are the most common treatments dentists perform [3, 4]. Restorative treatments are expensive for the individual and society. If left untreated, the disease might also cause severe pain, eating problems, social stigma, and reduced disability-adjusted life year (DALY) [1, 5]. The disease can be prevented and even reversed at its early stages by appropriate lifestyle adjustments [6–8]. Over the latest decades, access to regular dental care combined with increased awareness of the benefits of healthy teeth, increased systematic use of fluoride, and improved oral hygiene has led to significantly decreased mean caries prevalence, increased number of remaining teeth, and decreased prevalence of edentulous subjects in many parts of the world, including the Scandinavian countries [9–12]. However, the reduced mean caries prevalence hides a skewed disease distribution, where approximately 15-20% of the population remains with high disease activity and accounts for approximately 60% of caries development [13].

Successful treatment of diseases influenced by lifestyle, such as dental caries, cardiovascular diseases, and type-2 diabetes, relies on patient long-term compliance with risk factor management as well as on appropriate attention from the profession. For caries management, this means that dentists should identify an individual’s risk profile for disease development/progression and encourage patients to decrease their sugar intake, improve their oral hygiene, and increase fluoride use and frequency, all lifestyle actions that reduce cariogenic bacterial load. Therefore, caries risk assessment and risk factor profiling is recommended to ensure appropriate and early prevention and treatment of caries [14]. To individualize caries treatments and recall periods, most county councils in Sweden recommend that dentists use individual caries risk profiling [15]. This public dental care concept has recently been identified by Ito et al. [14] as a practical and effective strategy: “If people with higher risks can be identified and given improved intensive preventive care, this could offer both an efficient way of promoting individual and community oral health and a more economic use of health resources”.

Västerbotten County, located in northern Sweden, has required caries risk profiling for adults since 2002. Simultaneously, dentists have been urged to provide individualised preventive and non-operative caries measures in accordance with the minimally invasive caries concept and national guidelines [16, 17].

The aim of the present study was to evaluate the accordance between recommended treatment and a patient’s risk to develop caries by comparing caries development and clinical management of two patient groups: patients with no or low risk and patients with high risk for developing caries. Special attention was paid to caries preventive and non-operative measures. In addition, this study examines the reclassification of subjects into a lower or higher caries risk group. The study cohort comprised patients who regularly attended Public Dental Service clinics from 2005 to 2011 in Västerbotten County, Sweden.

## Methods

### Study design

This retrospective cohort study evaluates caries status and treatment from patient records for adults (25 years and older) who attended one of 33 Public Dental Service clinics in Västerbotten County, Sweden as recall patients from 2005 through 2011. The study is part of a larger cohort study on caries-lifestyle associations approved by the regional ethical review board in Umeå, Sweden.

### Caries scoring and risk assessment

Since 1999, an electronic system (T4) has been used for dental records at all Public Health Care clinics in Västerbotten. The system was developed by the Medical Insurance Agency (MIA) (Atlanta, USA), but is now marketed by Carestream Health (Toronto, Canada). The records include date of visit, oral (caries, periodontal, endodontic, soft tissues, plaque, etc.) and medical status, medication use, tobacco use, notes on specific examinations such as saliva and bacteria assessment, treatments and their costs, and type of recall system used. At each recall visit, a dentist or dental hygienist gave the patient a full oral examination. These examinations took place in fully-equipped dental offices and included at least bitewing x-rays in all patients where the approximal surfaces could not be inspected visually. The recall visits typically took place between six and 24 months after the initial visits. All 33 public dental health clinics use standardised examination routines for caries and periodontal disease. The dentists or hygienists conducted the caries examination using a mirror, probe, and x-rays, and scored lesions for enamel (initial) and dentine (manifest) caries [18]. The caries were classified either as Decayed, Missing, and Filled Teeth (DMFT) or as Decayed, Missing, and Filled Surfaces (DMFS).

In 2002, Västerbotten introduced mandatory assessment of risk – general, caries, periodontal, and technical risk (Table 1). Together, these form an overall risk score used when considering treatment plans and recall schedules. Risk assessment is repeated at every recall visit. Implementation of the risk assessment procedure in 2002 was accompanied by thorough education and repeated calibration exercises at each of the 33 clinics.

**Table 1:** **Overview of risk categories and criteria for risk assessment used in the County Council of Västerbotten, Sweden**

Risk category	Risk group 0 (no/low risk)	Risk group 1 (moderate risk)	Risk group 2 (high risk)
General	• No disease or medication affecting teeth or gums	• Disease and/or medication with possible effect on teeth or gums	• Disease or medication with significant effect on teeth and gums
	• Good oral hygiene	• Mediocre oral hygiene	• Poor oral hygiene
	• Adequate diet and intake frequency	• Partly inadequate diet	• Inadequate diet
		• Moderate dental anxiety	• Severe dental anxiety
		• Smoker or snuff user	• Heavy smoker (>20 cigarettes/day)
Caries	• No active enamel or dentin caries lesions	• 1-2 new caries lesions on caries prone surfaces	• ≥3 new caries lesions
		• New or moderate progression of enamel lesions	• Extensive progression of several enamel lesions
			• Lesions on non caries-prone surfaces
Periodontal	• Periodontal health	• Periodontitis experience	• Active periodontal disease with clinical and radio-graphic attachment loss
	• Gingivitis and/or supragingival calculus	• Localized periodontal problems/signs of local bone loss	• Subgingival calculus
	• Bleeding-free gingiva and no pocket exceeding >5 mm	• Bleeding and pocket depth of 5–6 mm	• Peri-implantitis
Technical	• Intact teeth or few restorations	• Single large restoration	• Several large restorations
	• Single root canal treatment of good quality	• Single restoration extending close to the pulp	• Several root canal treatments or root canal treatments of inadequate quality
	• Single crown or short bridge of good quality	• >1 root canal treatment of good quality	• Wisdom tooth requiring surgery
	• No or minimal abrasion of teeth	• Erupting wisdom tooth in the lower jaw	• Tooth grinding/TMD pain
		• Moderate abrasion of teeth/TMD pain	• Extensive erosion
		• Tongue/lip piercing	• Tongue or lip piercing with damaged teeth or mucosa
		• Crowns and/or bridges on healthy teeth with good occlusion	• Extensive teeth or implant supported constructions
		• Full or partial denture	

### Study population

In 2005, there were 196 998 adult inhabitants in Västerbotten County. Of these, 76 320 (39%) were registered as recall patients for complete dental care within the Public Dental Service. From this group, 42 276 had a regular dental examination in 2005 and 42% of these had a recall visit in 2011. In 2005, 35 896 adult patients (84.9% of all recall visits that year) had caries risk scored. In total, 44.3% of these patients were estimated to have no or low caries risk, 43% were estimated to have moderate caries risk, and 12.7% were estimated to have high caries risk. For the present study, 200 25–65 year-olds with high caries risk at the 2005 examination and with a recall visit in 2011 were randomly selected. This group was referred to as the “high caries risk group”. Selection was done to give equal proportions by sex, ten-year age groups, coast (more urban) areas, and inland (more rural) areas. An equally sized and proportionally balanced control group with no/low caries risk was randomly selected. This group was referred to as the “no/low caries risk group”. To ensure an 80% power of detecting difference at α = 0.05, the group sizes were based on expected annual caries incidence among high caries risk subjects in the Västerbotten population (mean 1.3 (SD 0.5) new surfaces/year). To account for a possible reduction in caries incidence over time, the group sizes were set to 200 subjects.

### Data retrieval from dental records

Three experienced dentists retrieved information on number of visits, type of personnel seen at the visit, caries risk score, medical condition and medication, use of tobacco, and type of treatment or counselling from patient records. Information on number of teeth, tooth status, clinic, and costs for operative and non-operative treatments were from data registers kept at the County Council. Calibration of the three dentists was achieved during the study protocol construction, and by evaluating and comparing an independent. *i.e.* not part of the study sample, selection of ten high caries risk and ten no/low caries risk. This strategy resolved any ambiguities in the protocol and among the examiners. Next, a template for interpretations was created and added to the review protocol. If any ambiguity appeared during the review, all three examiners discussed the issue to reach consensus.

### Data handling and statistical analysis

Data handling, descriptive analyses, and regression modelling were performed using SPSS version 20 software (SPSS Inc., Chicago, IL) and principal component analysis (PCA) using SIMCA P+, version 12.0 (Umetrics AB, Umeå, Sweden).

Estimated marginal means for caries prevalence and incidence were calculated among participants using general linear regression modelling (glm), including sex, age group, and clinic as covariates. Differences between means for the two caries risk groups were tested with Student’s *t*-test and among more groups (here clinics) by analysis of variance (ANOVA). Correlations between variables were calculated as Spearman or Fischer correlations depending on the distribution of observations. For categorical variables, distributions of numbers were tested using the Chi^2-test. P-values <0.05 were considered statistically significant.

Logistic regression was used to identify variables associated with (*i*) having a lower caries risk in 2011 if having a high caries risk in 2005 (n = 200) and (*ii*) maintaining a low/no caries risk in 2011 if having a low/no caries risk in 2005 (n = 200). The models included ten-year age group, sex, living region, and total numbers of scheduled visits to the dental office, and of visits that included counselling on tooth brushing with fluoridated toothpaste, the use of additional fluoride, dietary habits, and/or oral hygiene instruction.

Principal component analysis (PCA) was used to search for clustering among the study participants by caries risk group allocation or alteration in caries risk level from 2005 to 2011. The model searching for clustering of participants by caries risk group allocation included data for 2005 (ten-year age group, sex, living region, dental status, tobacco use, clinic, health condition, use of medication, and use of fluoride). The model searching for clustering of participants according to change in caries risk level from 2005 to 2011 also included information from 2011 on the variables listed for the logistic regression. Variables were autoscaled to unit variance before entering them into the PCA model, and clustering of subjects were displayed in a score-loading plot.

## Results

### Study group characteristics at baseline

**Figure 1:**
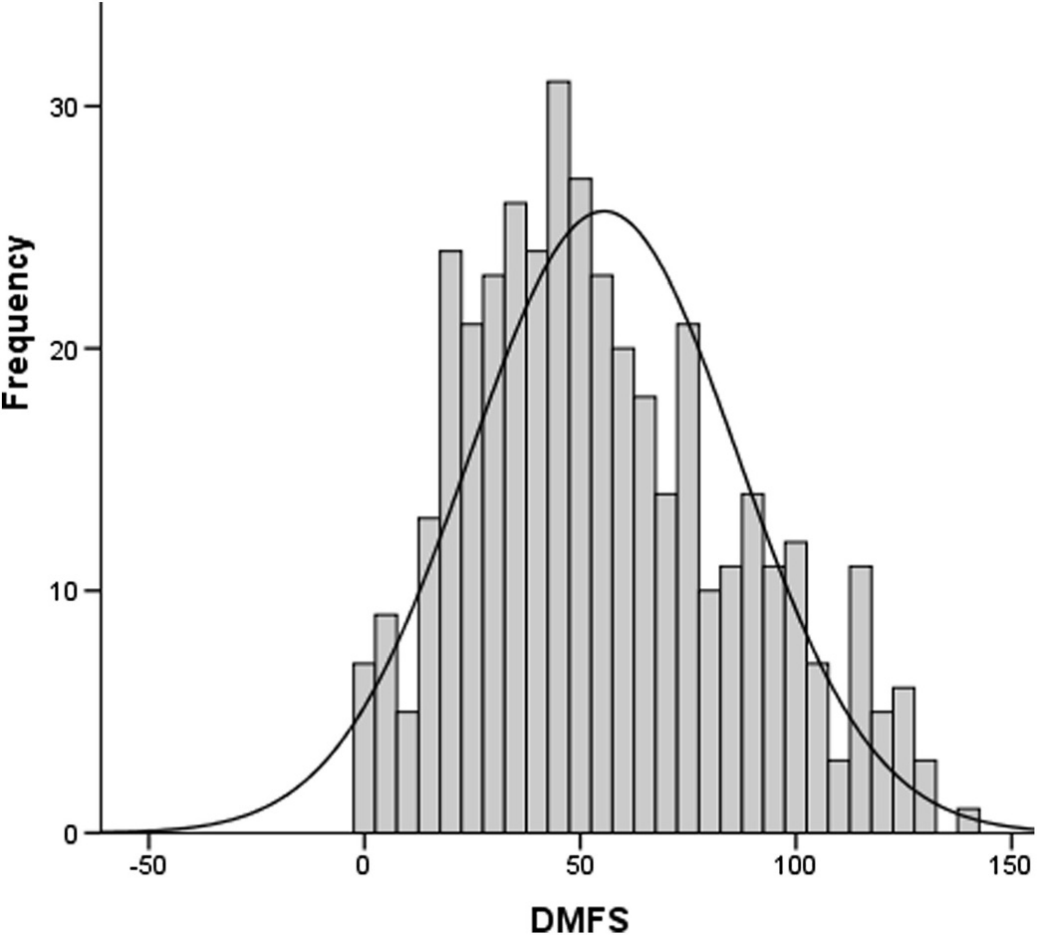
**Caries prevalence (DMFS**_**total**_ **) distribution.** The histogram involves all study subjects (n = 400) at baseline 2005. Mean DMFS was 55.5 (95% CI, 52.4-58.5). The solid line represents the fitted normal distribution curve.

**Figure 2:**
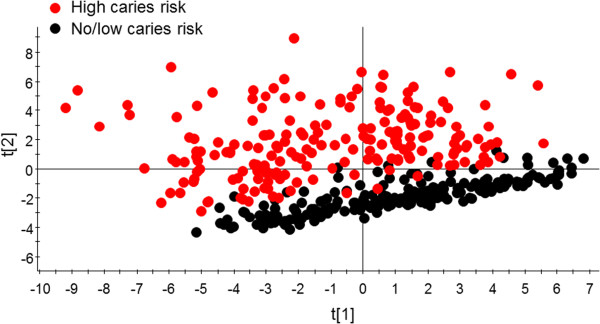
**PCA clustering of subjects with high versus no/low caries risk at baseline.** The PCA score plot shows modelling using baseline data. Model explanatory power (R^2^) and predictive power (Q^2^) by the two strongest components were 34.2% and 31.4%, respectively.

Two hundred randomly selected adults with a high risk for developing caries in the subsequent year were followed from 2005 to 2011 for caries incidence, caries preventive measures, non-operative caries treatments, and change in caries risk group allocation. Results were compared with data from a no/low caries risk group. The proportion of participants did not differ between the two groups with respect to self-reported disease, medication use, smoking use, or snuff (Swedish snus) use. However, sample selection in a ten-year age stratum led to slightly lower mean age in the high caries risk group compared with the low/no caries risk group at baseline: 43.1 (95% CI, 41.4-44.8) and 46.8 (95% CI, 45.3-48.3) years, respectively (Table 2). At baseline, the total number of teeth did not differ between the two groups, but the high caries risk group had significantly fewer intact teeth than the no/low caries risk (Table 2). Among all 400 participants, the distribution of caries prevalence (DMFS) was only slightly skewed to the right (Figure 1), a finding that supported the use of parametric analyses. Thus, overall mean DMFS was 55.5 (95% CI, 52.4-58.5). As expected, compared to those in the no/low caries risk group, significantly more decayed/missing/filled surfaces on all (DMFS_total_, p = 0.025) and approximal (DMFS_a_, p = 0.002) surfaces, more untreated lesions extending into the dentin (p < 0.0001), and more untreated secondary caries lesions (p < 0.0001) was seen in the high caries risk group (Table 2). Principal component analysis (PCA) employing baseline values distinctly separated high from no/low caries risk subjects as displayed by the two distinct swarms of differently coloured symbols (Figure 2).

**Table 2:** **Baseline (year 2005) characteristics of study participants according to caries risk group**

Variables (%, N)	No/low (***n***=200)	High (***n***=200)	***P***-value
	Caries risk group	Caries risk group	
Gender (%)	50%	50%	
Age (mean (95% CI))	46.8 (45.3-48.3)	43.1 (41.4-44.8)	0.001
Dental status (mean (95% CI))			
total number of teeth	27.4 (27.0-27.9)	27.6 (27.0-28.1)	0.770
number of intact teeth	14.4 (13.4-15.5)	12.6 (11.5-13.6)	0.015
Caries status (mean (95% CI))			
DMFS_total_	51.9 (47.8-56.0)	59.0 (54.4-63.4)	0.025
DMFS_approximal surfaces_	22.7 (20.5-24.8)	27.5 (25.3-29.8)	0.002
lesions in dentin (surfaces)	0.45 (0.30-0.60)	3.1 (2.6-3.6)	p < 0.0001
secondary caries (surfaces)	0.07 (0.03-0.10)	1.0 (0.8-1.3)	p < 0.0001
Health status (%)			
healthy	70.3	64.4	0.231
diseased	29.7	35.6	
Medication (%)			
non medicated	67.6	62.1	0.490
1-2 medicines	20.3	22.0	
≥ 3 medicines	12.1	15.8	
Tobacco use (%)			
no tobacco use	63.2	70.1	0.352
present smoker	12.1	12.4	
present snus user	23.1	16.9	
present smoker and snus user	1.6	0.6	
Preventive/non-operative measures (% treated)			
basic prevention^1^	48.5	57.0	0.089
additional fluoride	12.5	35.0	p < 0.0001
individual counselling on oral hygiene	21.0	21.5	0.903
Individual counselling on diet	0.5	6.5	0.005

^1)^Basic prevention implies population-based prevention and includes information about fluoridated toothpaste and brushing technique.

### Caries prevalence and incidence 2005 to 2011

**Figure 3:**
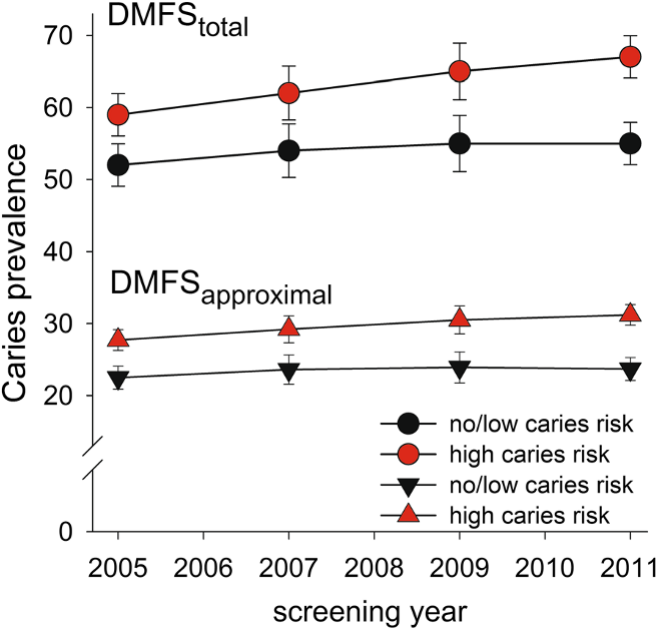
**Caries prevalence in the high and no/low caries risk groups over the seven-year study period.** Mean (95% CI) values are shown from 2005 through 2011 for all (DMFS_total_, upper panel) and approximal (DMFS_approximal_, lower panel) surfaces in the high caries risk (red) and no/low caries risk (black) groups, respectively.

Over the seven-year study period, caries prevalence (DMFS_total_) increased linearly in both caries risk groups, but with a steeper slope in the high-risk group (Figure 3). Thus, from 2005 to 2011, mean DMFS_total_ standardized for sex, age, and clinic increased by 7.8 (95% CI, 6.8-8.8) surfaces in the high risk caries group compared with 2.9 (95% CI, 1.9-3.9) surfaces in the no/low risk caries group (p < 0.001). These numbers do not include secondary caries. A similar pattern, although less pronounced, was seen for caries prevalence on approximal surfaces, DMFS_approximal_ (Figure 3).

**Figure 4:**
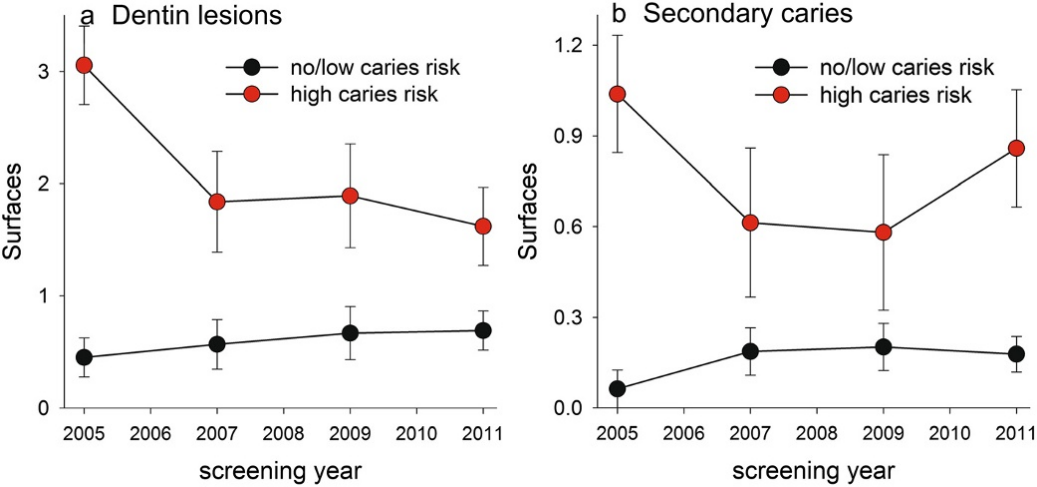
**Caries incidence in the high and no/low caries risk groups over the seven-year study period.** Data are shown as mean (95% CI) for **a)** new primary caries lesions reaching into the dentin and **b)** new secondary caries lesions.

Incidences of primary and secondary dentin lesions between treatment sessions, which averaged 13 months for the high and 18 months for the no/low caries risk group, were significantly higher in the high caries than in the no/low caries risk group at all time points (Figures 4a,b). However, the 2005 mean numbers for incident primary dentin lesions were lower for all subsequent years in the high caries group, and the 2007 and 2009 mean numbers for incident secondary caries lesions were lower, but this trend had reversed by 2011 (Figure 4b). In contrast, incident caries (primary and secondary) were virtually stable over time in the no/low caries risk group (Figures 4a,b). Paralleling the higher caries incidence in the high caries risk group, their mean number of intact teeth decreased by 1.4 teeth over the seven-year study period compared to 0.4 teeth in the no/low caries risk group (p < 0.001, data not shown).

### Caries risk assessment

**Figure 5:**
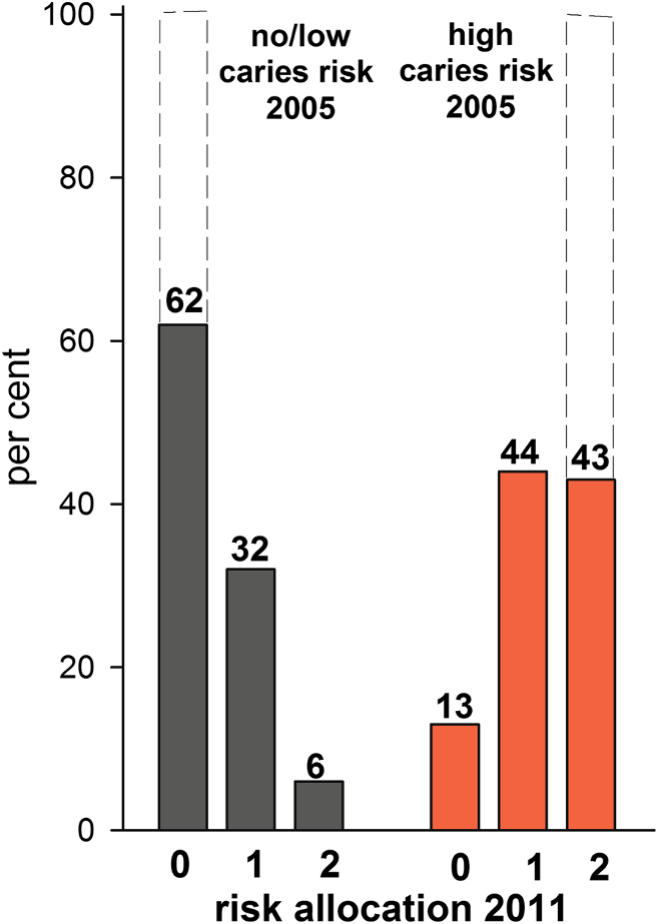
**Caries risk score allocation at the end of the seven-year study period.** Filled bars show proportion (%) of subjects allocated to various caries risk scores (0 = no/low risk, 1 = moderate risk, and 2 = high risk) in 2011. This should be compared with baseline in year 2005, when 100% of the subjects were allocated to no/low caries risk or high caries risk, respectively (here indicated by bars drawn with dotted lines).

**Figure 6:**
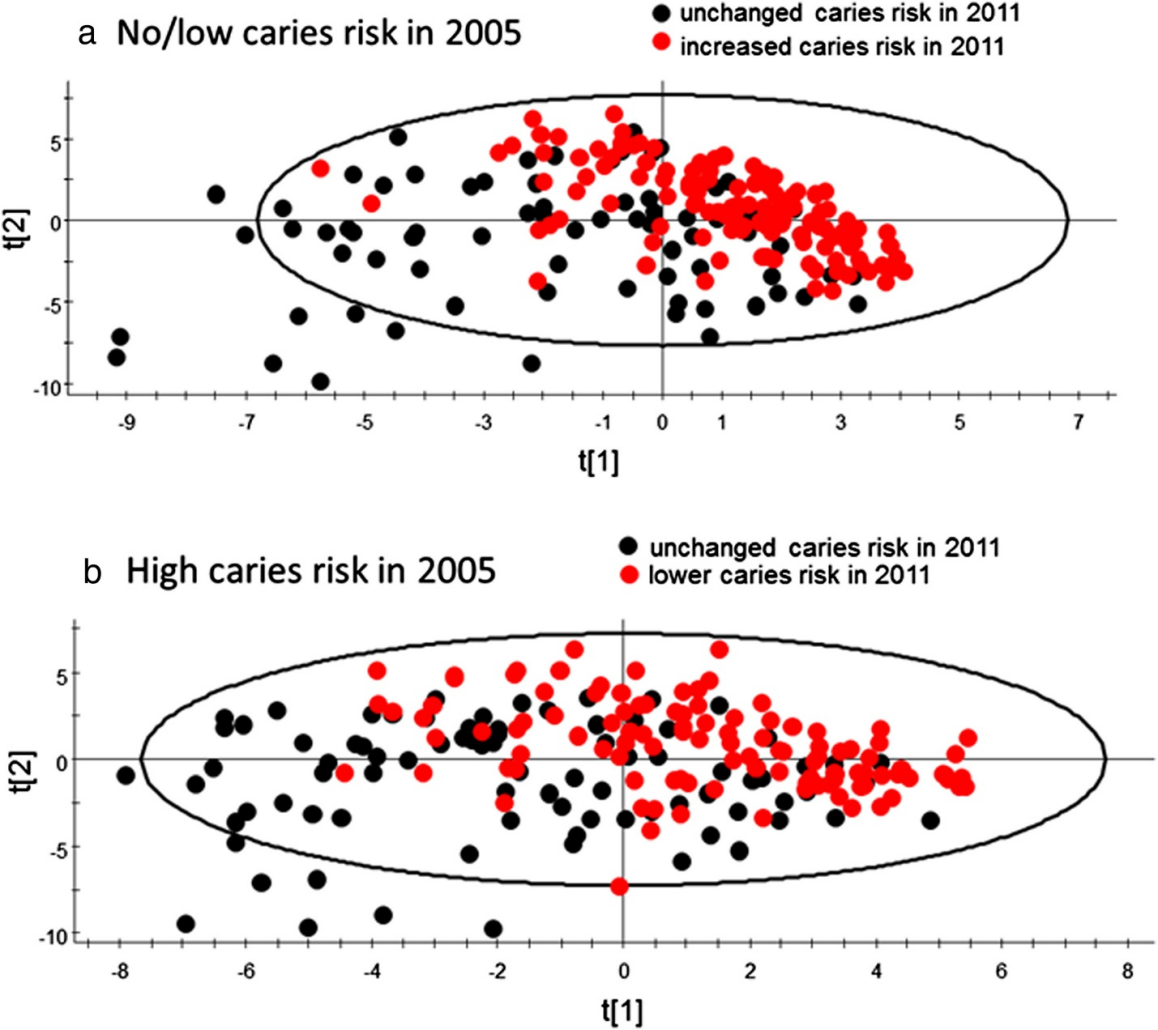
**PCA clustering of subjects by alteration in caries risk scoring over the seven-year study period.** PCA score plots clustering subjects who **(a)** did or did not maintain their caries risk score over the seven-year study period if having low/no risk in 2005 or **(b)** who did or did not improve their caries risk score over the seven-year study period if having high risk in 2005. The model explanatory power (R^2^) was 33.5% and 32.4% for **a)** and **b)**, respectively, and the predictive power (Q^2^) was 22.1% and 24.0% for **a)** and **b)**, respectively.

By 2011, 43% of the 2005 high caries risk group still had a high caries risk score, 44% had their baseline score lowered to moderate risk, and 13% had their baseline score lowered to no/low risk (Figure 5). In comparison, 32% of the no/low caries risk subjects in 2005 had increased their caries risk to a moderate risk and 6% to a high risk. PCA modelling clustered those who had maintained a no/low caries risk scoring from 2005 to 2011 separate from those who had increased their risk to moderate or high (Figure 6a). Similarly, PCA separated those who remained with a high caries risk from 2005 to 2011 from those who had a risk in 2011 lower than in 2005 (Figure 6b). The proportions of subjects who remained with a no/low caries risk score from 2005 to 2011 varied among clinics, from 90 to 24% (p = 0.090 for overall testing between clinics). Similarly, the proportions with a lower caries risk score in 2011 if scored with a high caries risk in 2005 varied between clinics, from 89 to 18% (p = 0.057 for overall testing between clinics).

### Preventive and non-operative treatment measures

At baseline, it was documented that approximately half of the participants received counselling on the use of fluoridated toothpaste (basic prevention), but no significant difference between the two groups was found (Table 2). Furthermore, 35% of the high-risk subjects were, according to records, told to use additional fluoride compared to 12.5% in the low/no risk group (p < 0.0001).

Over the seven-year study period, 12.3% of the 400 studied subjects were never counselled on basic preventive measures, 43.0% were never recommended to use any type of fluoride besides that in toothpaste, 44.5% were never counselled on oral hygiene, and 90.5% were never counselled on dietary habits. Still, counselling on caries preventive measures was significantly more frequently documented in the high than in the no/low caries group (Table 3). The subjects in the high caries risk group were given advice on how to supplement their fluoridated toothpaste use with other fluoride treatments, how to improve their oral hygiene, and how to improve dietary habits. On average, these suggestions were presented 1.8, 1.2, and 0.2 times, respectively (Table 3).

**Table 3:** **Number of visits to the dental clinic and counselling opportunities over the seven-year study period (2005–2011)**

Variables (N)	No/low	High	***P -***value
	Caries risk group	Caries risk group	
Number of visits to the dental clinic	11.2 (10.0-12.4)	20.4 (19.2-21.6)	p < 0.0001
Number of visits to a dentist	7.5 (6.4-8.6)	16.2 (15.1-17.4)	p < 0.0001
Number of visits to the dental hygienist	3.7 (3.18-4.2)	4.1 (3.6-4.6)	0.296
Number of acute visits to the clinic	2.3 (1.7-3.0)	4.4 (3.7-5.0)	p < 0.0001
Annual cost for dental treatments (SEK)	1 192	2 677	p < 0.0001
Recall period (months)	17.5 (17.1-17.9)	13.4 (13.1-13.7)	p < 0.0001
DMFS_total_ 2005-2011	2.9 (1.9-3.9)	7.8 (6.8-8.8)	p < 0.0001
Counselling			
basic prevention package^1^	2.30 (2.08-2.52)	2.77 (2.54-2.99)	0.004
increased fluoride exposure	0.79 (0.59-0.99)	1.84 (1.64-2.04)	p < 0.0001
individual oral hygiene instruction	0.85 (0.69-1.01)	1.15 (0.99-1.31)	0.009
individual dietary habit information	0.06 (0.00-0.13)	0.21 (0.15-0.28)	0.001

^1)^Recommendation to use fluoridated toothpaste and basic information on oral hygiene and diet.

Data are presented as mean (95% CI) adjusted for sex, age, and clinic for the seven-year study period.

Logistic regression modelling revealed that an improved caries risk score in 2011, compared to their 2005 score, was associated with more counselling on tooth brushing with fluoridated toothpaste, whereas older age at baseline was associated with less likeliness of an improvement (Table 4, section A). Maintenance of a low/no caries risk score from 2005 to 2011 was positively associated with increasing age and borderline associated with the number of counselling sessions and instruction on oral hygiene, whereas being a man was strongly associated with not maintaining a low/no caries risk score over the seven-year period (Table 4, section B). The number of sessions with any type of preventive or non-operative caries measures correlated with the number of visits to a dental hygienist (correlation coefficients 0.455 and 0.324, p *<* 0.001), whereas the total number of visits to the clinic was strongly correlated with number of visits to a dentist (correlation coefficients 0.920, p < 0.001) (data not shown).

**Table 4:** **Logistic regression Odds ratio (β-coefficient) and 95% CI for (A) a lower caries risk score in high risk subjects, or (B) maintain a low caries risk score in 2011 compared to at baseline (2005) and 2011; reference group in parenthesis**

Variables retained in model^1,2^	β-coefficient	95% CI for β	***P***-value
**(A) Odds ratio to have a lower caries risk score in 2011 if high risk in 2005 (n = 200)**			
Total number of visits to the dental office	0.92	0.89-0.96	<0.001
Counselling on tooth brushing with fluoridated toothpaste (lowest number odds = 1)	1.44	1.11-1.86	0.006
Counselling and training on tooth cleaning (lowest number odds = 1)	0.59	0.42-0.82	0.002
**(B) Odds ratio to maintain a low/no caries risk score from 2005 to 2011 (n = 200)**			
Sex (women odds = 1)	0.34	0.17-0.68	0.002
Age group (youngest ten-year age group odds = 1)	1.05	1.02-1.09	0.005
Total number of visits to the dental office	0.86	0.80-0.91	<0.001
Counselling and training of tooth cleaning (lowest number odds = 1)	1.39	0.96-1.99	0.080^2^

^1)^The basic model included sex, ten-year age group at 2005, living region, and total number of visits to the dental office, and numbers of visits with counselling on tooth brushing with fluoridated toothpaste, additional fluoride, dietary habits, and/or oral hygiene instruction. Variables not shown in the table did not meet the criteria of a probability <0.10 in the final step. The reference (odds ratio = 1) is to A) have no improvement and B) to not have a maintained low/no risk.

^2)^Models restricted to one counselling type at a time (covariates sex, ten-year age group at 2005, living region, and total number of visits to the dental office) confirmed the results from the basic model and reached statistical significance for counselling and training of tooth cleaning (p = 0.031) in section B.

### Number of visits and costs

On average, the length of the recall period (i.e., months until next full examination) was once every 12 months in the high caries risk group and once every 18 months in the no/low caries risk group. Thus, the mean recall period was 13.4 (95% CI, 13.1-13.7) and 17.5 (95% CI, 17.1-17.9) months for the respective groups (p < 0.0001, Table 3). These recall intervals were stable over the seven-year study period in both groups (data not shown). In total, the high caries risk group paid nearly twice as many visits to the dental clinic as the no/low caries risk group over the seven-year study period (i.e., 20.4 versus 11.2 visits, p < 0.0001), including a significantly higher number of emergency visits (Table 3). Correspondingly, the average cost for dental treatments was 2.24 times higher in the former compared to the latter group (p < 0.0001). The difference in number of visits was explained by a higher number of visits to a dentist, as the number of visits to a dental hygienist did not differ between the two groups (Table 3).

## Discussion

The present study evaluated caries development and clinical management of adult patients classified with high risk and activity of caries and compared these findings with subjects classified with low/no risk and activity of caries. The study was performed in a county with organized dental care that provided guidelines for prevention and non-operative treatments in caries management. Three main findings were identified: (*i*) subjects at high risk of developing caries continued to develop disease at a higher level than low/no risk subjects, and nearly 50% of these remained high risk despite significantly more frequent visits to the dental clinic than those with low risk; (*ii*) preventive measures were according to records at an unexpectedly low level for high risk subjects and only marginally different in type and amount for low risk subjects; and (*iii*) preventive measures, especially the recommendation to use fluoridated toothpaste, were associated with a reclassification of high caries risk subjects into moderate or low risk. Although the use of fluoridated toothpaste has been established to improve caries prevention in children and adolescents [19], in adults there is little evidence that such measures are helpful. Therefore, it was noteworthy that counselling on tooth brushing with fluoridated toothpaste, even if far from optimal, was associated with a lowering of the risk score in those originally scored with a high risk.

In Västerbotten, the Public Dental Service provides regular dental care to virtually all residents aged 20 and under and nearly 40% of the adult residents. Dental care is free up to the age of 20 and subsidized for anyone 20 years and older. Most patients attend the same dental clinic on a long-term basis. In general, recall visits are between one and two years. These characteristics, as well as the overall socio-economic profile, are not considered to differ substantially between adults treated by the Public Dental Service or by private clinics [12, 20]. Thus, a strength of this study is that its subjects represent the general population of the area. It has previously been found that behaviours and attitudes towards oral disease prevention differ between clinics [21], a finding that was supported in the present study by the wide variation in proportion of patients reducing their high or maintaining their low/no caries risk level. Thus, another strength was that all clinics in the county were represented, reducing a potential impact from systematic errors. Furthermore, means were standardized for clinics to adjust for the uneven proportion of subjects from the different clinics after sample selection. A weakness of the study relates to the retrospective evaluation of patient records as this approach could result in underreporting of treatments and counselling. In addition, the retrospective design may be a limitation since ambiguities could not be resolved with the caregiver(s); however, this situation could be seen as a strength because treatments and recordings were not intentionally or unintentionally adjusted to a study situation. An additional potential source of error relates to the validity of the caries scoring, and especially that of secondary caries. It cannot be excluded that scoring of secondary caries is biased by inclusion of marginal fractures of restorations. Hence, the increase in secondary caries from 2009 and 2011 might, at least partly, reflect an increased number of restorations and restoration fractures. We do, however, not think this is a major source of error based on the facts that the digital recording system has a specific code for restoration fracture, and that during the years 2005 to 2009 incident secondary caries decreased though the number of restorations increased steadily, and there is no reason recording habits would change significantly over a few years in a stable staffing.

The goal for the national Public Dental Service, which is also the goal for Västerbotten, is to achieve and maintain good oral health in all citizens [17]. The primary guideline is that all patients should be knowledgeable on basic oral hygiene procedures and on the benefit of using fluoridated toothpaste. In addition, a high-risk strategy targeting the individual risk profile should be used in highly diseased individuals or individuals susceptible to disease. Therefore, it was surprising that information on tooth brushing and use of fluoridated toothpaste was documented in only half of the 400 participants and even more surprising that the proportions receiving such information, or any other preventive counselling, did not differ substantially between the high and low/no caries risk groups at baseline or over the entire seven-year period. It was also unexpected that the number of visits to a dental hygienist did not differ by the two study groups. There are several theoretical explanations for this. For example, information may have been given but not recorded and patients who had such information before 2005 may have already been practising good oral hygiene and using fluoridated toothpaste. However, if the latter were true, one would expect to find this documented at least in the high-risk group. This was rarely the case. Another possible explanation relates to dentists’ and dental hygienists’ lack of trust that their patients will follow through with prevention protocols. For example, Sbaraini et al. found that many Australian dentists considered some patients too “unreliable” to benefit from prevention counselling, so tangible restorative treatment offered “value for money” [22]. Whether this reflects the situation in Sweden can only be speculated on, but given that both countries have a similar system for dental education and care, it cannot be excluded. Still, the overall conclusion is that compliance with the guidelines on caries prevention and treatment are poorly followed, but this conclusion needs further study.

The present findings of a seemingly non-optimal compliance with the Swedish guidelines for caries treatment and prevention and the continuous need for frequent visits to the clinic for high risk patients is fully in line with Rindal et al.’s findings [23]. A portion of the limited effect of counselling and treatment certainly relates to limited compliance among patients and eventually shortcomings in treatment efficacies, such as reported from randomized clinical trials with high-risk strategies [24, 25]. The present study does not allow for a distinction between the impact of compliance among patients and caregivers. Such a distinction would help researchers and dentists design strategies that improve disease outcome. As with high-risk patients, low/no risk patients should have a level of treatment commensurate with their risk and prevention goals. It cannot be evaluated whether the present results reveal an over-treatment in low risk subjects, but the lack of difference in treatment intensity in the two groups opens the question.

Disease risk assessment is a generally accepted way to identify patients who need intensified care versus those who do not. The concept, however, relies on the accuracy of the risk assessment instrument and that appropriate treatments are practiced [8]. None of the presently suggested markers for caries risk have been shown to have satisfactory sensitivity and specificity irrespective of whether clinical or biological parameters were tested. This lack of specificity and sensitivity may reflect the fact that the optimal combination of markers has yet to be identified, and that caries patients represent distinct subgroups where lifestyle factors are more influential for some patients and biological susceptibility is more prevalent in others [26]. The risk scoring system used at the study clinics rely mainly on disease activity, even though general disease, medication, and dentist’s opinion are considered. Apparently, this system was sufficient to distinguish between patients with low or high caries risk, as those scored with high risk both have higher caries prevalence and a higher caries incidence over the seven-year period. However, the risk scoring system does not guide the design of the individual treatment plans as one based on individually-assessed biological and behaviour risk factors.

## Conclusions

This study formulated two main conclusions: (*i*) adult patients with high or no/low caries represent different populations, that each contain distinct subpopulations, those who improve/impair or maintained their caries risk and disease progression. These groups need different strategies in disease treatment; and (*ii*) some preventive measures and non-operative treatments were associated with improvements in caries risk and maintenance, but the extent to which such treatments were given to high caries risk subjects was unacceptably low and not in line with present guidelines. Future research should evaluate what aspects influences dental caregivers to provide and succeed in caries preventive treatments also in patients at high risk to develop disease. In addition, future studies should examine how to improve implementation of preventive treatments as well as aim at separating the estimation of the effectiveness of such treatments from the effects of compliance.

## Authors’ information

US is responsible for development and quality control in the Public Dental Health organization in the County of Västerbotten. IJ is professor in Cariology with vast experience in epidemiology. KSG (DDS, PhD) is experienced in caries treatment in adult patients and in designing and conducting clinical trials.
